# A key role for hepatitis C virus NS5A serine 225 phosphorylation revealed by super-resolution microscopy

**DOI:** 10.1038/s41598-025-93812-w

**Published:** 2025-03-20

**Authors:** Niluka Goonawardane, Chunhong Yin, Grace C Roberts, Carsten Zothner, Mark Harris

**Affiliations:** 1 School of Molecular and Cellular Biology, Faculty of Biological Sciences, and Astbury Centre for Structural Molecular Biology, University of Leeds, Leeds, LS2 9JT UK; 2 Present Address: Infectious Disease Control Institute, Shandong Center for Disease Control and Prevention, Shandong Provincial Key Laboratory of Infectious Disease Prevention and Control, Jinan, 250014 Shandong People’s Republic of China

**Keywords:** Hepatitis C virus (HCV), Non-structural protein 5A (NS5A), RNA replication, Sub-genomic replicon, Phosphorylation, Expansion microscopy (ExM), Super-resolution microscopy, Hepatitis C virus, Virology

## Abstract

NS5A is a multi-functional phosphoprotein that plays a key role in hepatitis C virus (HCV) genome replication and assembly. The consequences of NS5A phosphorylation for HCV biology remain largely undefined. We previously identified serine 225 (S225) as a major phosphorylation site within the low complexity sequence 1 (LCSI) of NS5A and used a phosphoablatant mutant (S225A) to define the role of this phosphorylation event in genome replication, NS5A-host interactions and sub-cellular localisation. In this study, we investigate this further by raising an antiserum to S225 phosphorylated NS5A (pS225). Western blot analysis revealed that pS225 was predominantly in the hyper-phosphorylated NS5A species. Using a panel of phosphoablatant mutants of other phosphorylation sites in LCSI, we obtained evidence that is consistent with bidirectional hierarchical phosphorylation initiated by phosphorylation at S225. Using super-resolution microscopy (Airyscan and Expansion), we revealed a unique architecture of NS5A-positive punctae in HCV-infected cells; pS225 was present on the surface of these punctae, close to lipid droplets. Although S225 phosphorylation was not specifically affected by treatment with the NS5A-targeting direct acting antiviral agent daclatasvir, this resulted in the condensation of NS5A-positive punctae into larger structures, recapitulating the S225A phenotype. These data are consistent with a key role for S225 phosphorylation in the regulation of NS5A function.

**Supplementary Information:**

The online version contains supplementary material available at 10.1038/s41598-025-93812-w.

## Introduction

Hepatitis C virus (HCV) infects an estimated 58 million people and frequently results in chronic liver disease, cirrhosis and hepatocellular carcinoma^1,2^. HCV was recently reclassified as the species *Hepacivirus hominis*^3^ and is a member of the *Hepacivirus* genus within the *Flaviviridae* virus family. HCV has a ~ 9.6 kb positive-stranded RNA genome with 5′ and 3′ untranslated regions (UTRs) flanking a single open reading frame encoding a ~ 3,000-residue polyprotein precursor^4^. This is co- and post-translationally processed by host and viral proteases to yield four structural proteins (core, E1, E2, and p7) and six non-structural proteins (NS2, NS3, NS4A, NS4B, NS5A, and NS5B). The NS proteins play a number of roles during the HCV life cycle^5^. NS3-NS5B are necessary and sufficient for HCV genome replication in association with multiple-membrane vesicles (MMV)^6^, whose formation is induced by NS4B and NS5A. MMVs compartmentalise genome replication complexes (RC)^7–9^ shielding them from host defences and concentrating components to enhance replicative efficiency. Polyprotein processing and genome replication are the targets of recently developed direct-acting antivirals (DAAs), used in combination therapies to effectively treat HCV infection. DAAs target the NS3/4A protease, NS5A, and the NS5B RNA-dependent RNA polymerase and have revolutionised HCV therapy^10^. Daclatasvir (DCV) exemplifies a class of DAAs proposed as potent inhibitors of NS5A^11,12^. However, the mode(s) of action for these compounds remains obscure as it is unclear which of the myriad NS5A functions are inhibited.

**Fig. 1:**
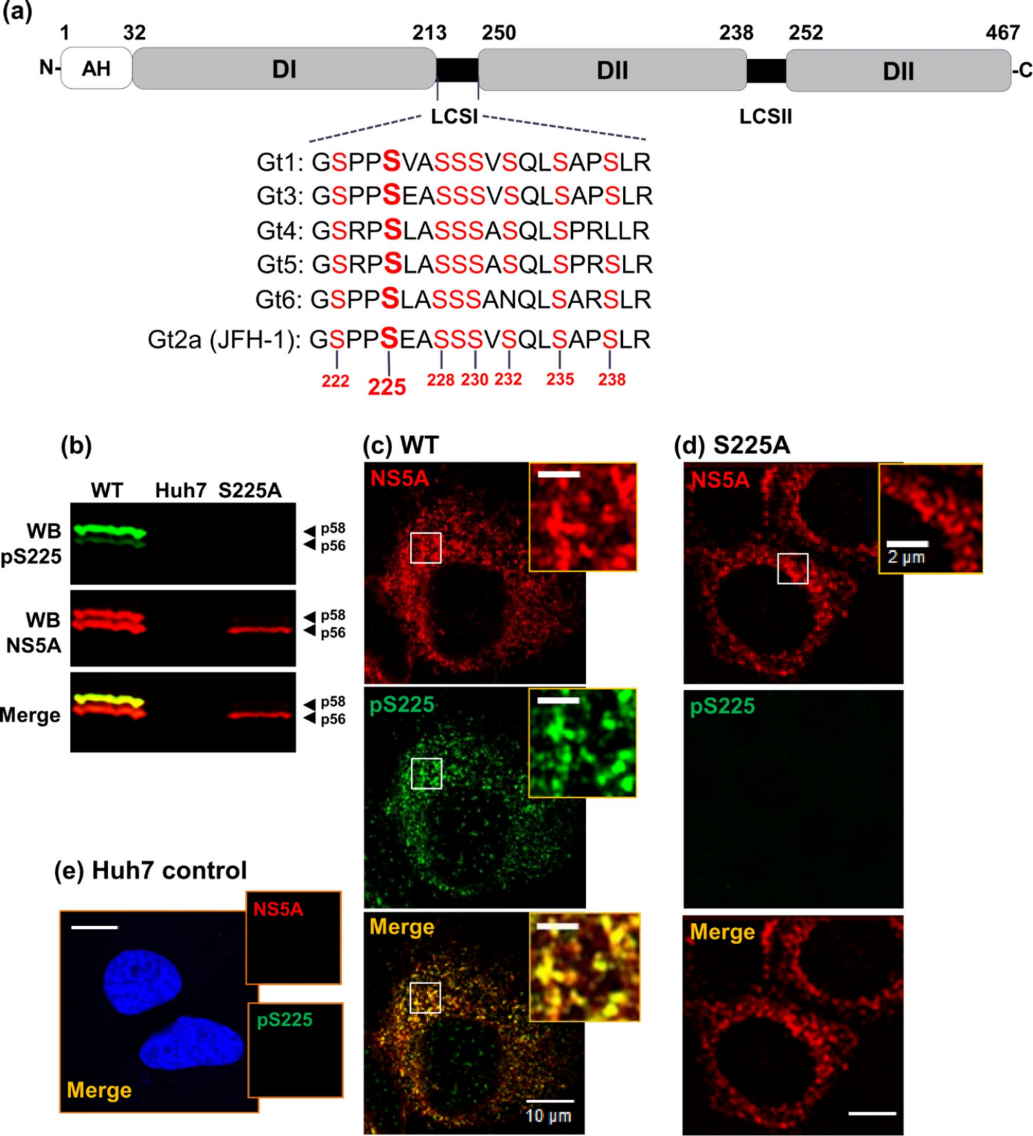
Validation of pS225 antiserum. (**a**) Schematic representation of the NS5A structure. The three domains (I-III), linking low complexity sequences (LCSI and II), and the membrane anchoring amphipathic helix (AH) are illustrated with peptide sequences of the LCSI region from six major HCV genotypes. Numbers indicate amino acid positions in the JFH-1 genotype 2a NS5A sequence. (**b**) Specificity of the pS225 antibody. Lysates from control Huh7 cells, or Huh7 cells electroporated with either wildtype (WT) or S225A mJFH-1 RNA analyzed by western blotting with either rabbit anti-pS225 (green), or sheep anti-NS5A (red) polyclonal antisera. (**c**-**e**) Huh7 cells electroporated with WT (**c**) or S225A (**d**) mJFH-1 RNA were analysed by immunofluorescence with anti-pS225 or anti-NS5A antisera. (**e**) Uninfected Huh7 cells were used as negative control. Scale bars: 10 μm and 2 μm (insets).

NS5A is a multifunctional protein comprising three domains (DI-III) and an N-terminal amphipathic helix anchoring it to cytoplasmic membranes (Fig. 1A). The structure of DI has been determined^13–15^, in contrast DII and DIII are intrinsically disordered, with elements of secondary structure^16^. The domains are linked by low complexity sequences (LCS) I and II (Fig. 1A), LCSI is serine-rich, and LCSII is proline-rich. All three domains bind the HCV 3′ UTR^17^: DI functions in both genome replication and virus assembly^18,19^, DII is exclusively involved in genome replication^20^ and DIII plays a critical role in virion assembly^21^. NS5A associates with lipid droplets (LDs) in infected cells^22^, where it is postulated to link the processes of genome replication and virus assembly^18,19^. NS5A also interacts with many cellular proteins^23,24^, modifying the host cell environment to favour the virus. This likely requires a subset of NS5A distinct from that involved in genome replication or virus assembly.

The effects of phosphorylation on the conformation and interactions of NS5A have been extensively studied but remain ambiguous. NS5A manifests as two species with different mobility on SDS-PAGE termed basally phosphorylated (apparent molecular weight 56 kDa), and hyper-phosphorylated (58 kDa). Of note, the cell culture infectious JFH-1 (genotype 2a) HCV isolate contains an 18-residue insertion in DIII compared to genotype 1b (14) and the two JFH-1 species migrate at ~ 63 and 65 kDa. However, for consistency with the literature, they are referred to herein as p56 and p58. NS5A is phosphorylated on multiple serine and (to a lesser extent) threonine residues^25–30^. Whereas p56 is reportedly phosphorylated within DII and DIII, multiple phosphorylation events within the serine-rich LCSI result in the production of p58 (Fig. 1A).

Our previous studies identified a key role for S225 phosphorylation – the S225A phosphoablatant mutant had a dramatic phenotype: a loss of p58, a 10-fold reduction in JFH-1 genome replication, a loss of interactions between NS5A and a range of host proteins, and a perinuclear-restricted localisation^23,31^. This phenotype prompted us to further investigate the role of S225 phosphorylation. In this study, to complement the analysis of S225 mutants, we raised an antiserum to pS225-NS5A and used this to probe the functions of S225-phosphorylated wildtype NS5A. We demonstrate that pS225 is predominantly present in p58 and provide further evidence that pS225 is a ‘priming’ phosphorylation event that drives subsequent phosphorylation at other serine residues within LCSI. NS5A is located throughout the cytoplasm in punctae, using super-resolution microscopy approaches, we reveal a new and detailed architecture of these NS5A-positive structures: in HCV-infected cells pS225 is not uniformly distributed but is instead present at distinct locations on the surface of the punctae. Super-resolution analysis also revealed that treatment with DCV results in profound disruption to the architecture of these clusters, which become larger, less ordered and more diffuse. The same morphology was observed in the context of the S225A phosphoablatant mutant. The combination of the pS225 antiserum and super-resolution microscopy thus revealed unique insights into the role of NS5A phosphorylation and are consistent with its key role in the regulation of NS5A function during the HCV lifecycle.

## Results

### Characterisation of an antiserum specific for S225 phosphorylated NS5A

We previously demonstrated a role for S225 phosphorylation in HCV genome replication, and further showed that this was mediated via interactions of NS5A with a defined subset of cellular factors^23,31^. A caveat to these studies was the necessary dependence on an interpretation of data based on the analysis of the phenotype of S225 mutants (S225A phosphoablatant or S225D phosphomimetic). To address this, we raised a rabbit polyclonal antiserum specific for the S225 phosphorylated form of NS5A using an appropriate phosphopeptide as immunogen (CARGSPP**pS**EASSS). The resulting anti-pS225 antiserum was validated by western blotting (Fig. 1B). When used to probe lysates from Huh7 cells infected with wildtype HCV (mJFH-1), this antiserum predominantly detected the p58 NS5A species, illustrated by the merged image of pS225 (green) and total NS5A (red) signals. This was consistent with previous observations that the S225A phosphoablatant mutant resulted in a loss of p58^31^. The antiserum also exhibited weak reactivity with p56, this may be due to incomplete affinity purification of the phospho-S225 reactive antibodies, or the presence of a low level of pS225 in the p56 basal phosphorylated form. The latter is most likely as the pS225 antiserum exhibited no reactivity against the S225A mutant (Fig. 1B).

We next validated the use of this antiserum for immunofluorescence analysis by confocal microscopy. Huh7 cells stably harbouring a wildtype sub-genomic replicon (SGR) were stained for total NS5A (red) and pS225 (green) (Fig. 1C). The results showed that in wildtype SGR-harbouring cells pS225 distribution significantly co-localised with NS5A staining. However, consistent with the western blot analysis, the co-localisation was incomplete as NS5A reactivity was observed that did not colocalise with pS225. The specificity of the pS225 antiserum was further confirmed by a lack of cross-reactivity with either the S225A phosphoablatant NS5A or control cells (Fig. 1D,E). Taken together, these data validated the use of this unique reagent as a tool to investigate the properties of S225-phosphorylated NS5A in more detail.

### Evidence for a role of S225 phosphorylation in initiating hierarchical phosphorylation in LCSI

A number of lines of evidence point towards a hierarchical or sequential phosphorylation cascade across LCSI. These include the conservation and spacing of serine residues, and mass spectrometric evidence for multi-phosphorylated species corresponding to LCSI^26,29,32^. Additionally, use of pS232, pS235 and pS238-specific antisera showed that pS232 primed phosphorylation at S235 and S238^27,28^, suggesting that S232 was the primary phosphorylation site leading to a hierarchical phosphorylation of other serines within LCSI. However, these studies did not directly address the potential role of S225 phosphorylation and we hypothesised that this might precede phosphorylation of S232 to initiate the hierarchical phosphorylation cascade.

**Fig. 2:**
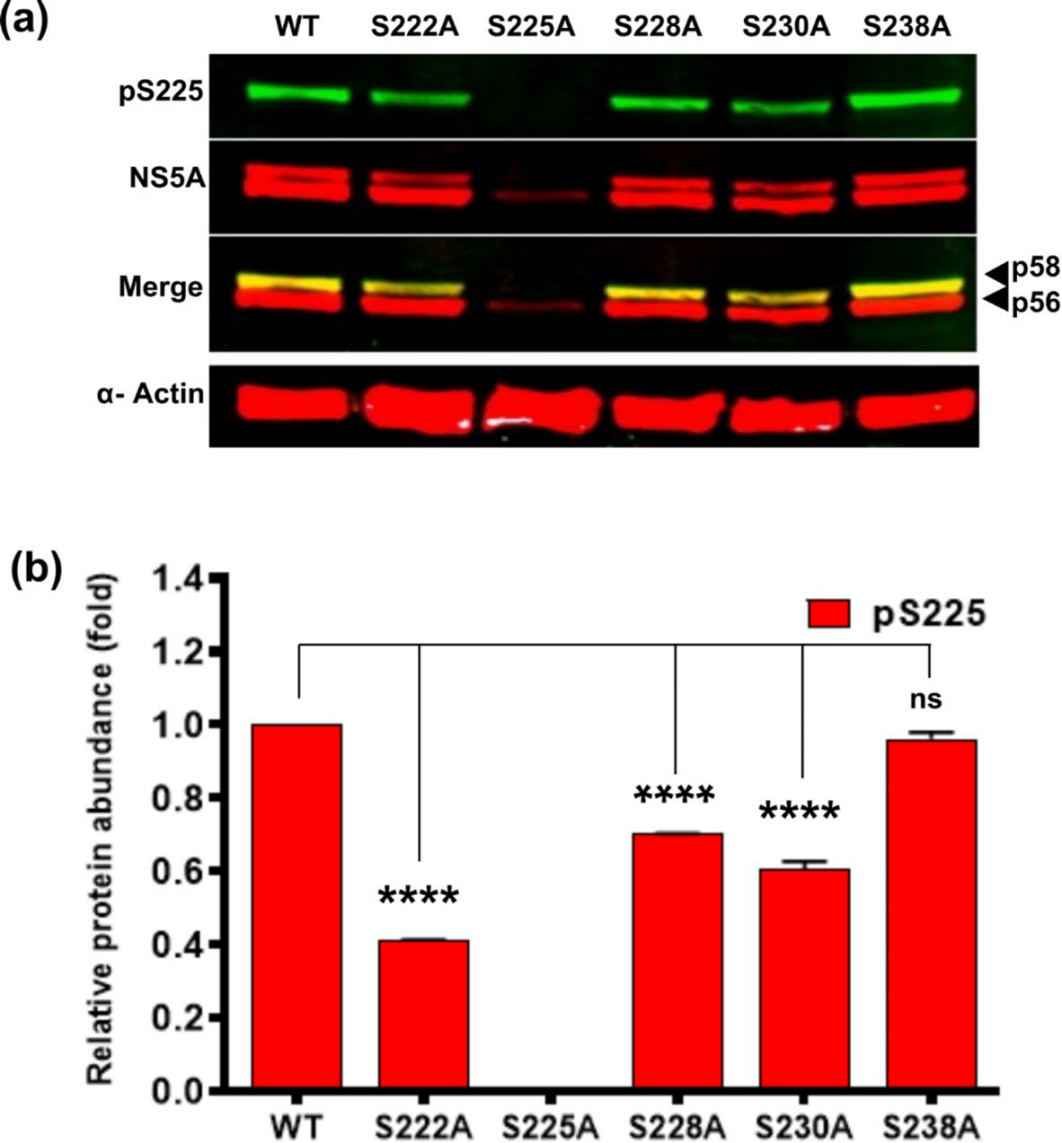
S225 phosphorylation is independent of other phosphorylation events in LCSI. Huh7 cells were electroporated with the indicated mJFH-1 WT and mutant RNAs. (**a**) Lysates were analysed by western blotting for pS225, total NS5A and α-actin. (**b**) Quantification of pS225 (red) abundance from multiple western blots (*n* = 3). Values are mean ± SE. **** *P* > 0.0001.

To provide evidence for this hypothesis, we used western blot analysis to examine the presence of pS225 in cells infected with mJFH-1 phosphoablatant mutants: S222A, S225A, S228A, S230A and S238A. We were unable to interrogate the remaining phosphoablatant mutants as they were replication-inactive^26^. As shown in Fig. 2A, the only mutant which completely abolished pS225 reactivity was S225A itself, although pS225 levels were reduced by between 30 and 60% in 3 of the other mutants (S222A, S228A and S230A) (Fig. 2B). This suggests that S225 phosphorylation was independent of other phosphorylated serines in LCSI but exhibited a partial dependence on the surrounding amino acid sequence. This is consistent with the hypothesis that phosphorylation of S225 primes hierarchical phosphorylation of other serines in LCSI.

Of note, S225A exhibited low overall levels of NS5A, consistent with the previously observed 10-fold reduction in HCV genome replication by this mutation^23,26,31^.

**Fig. 3:**
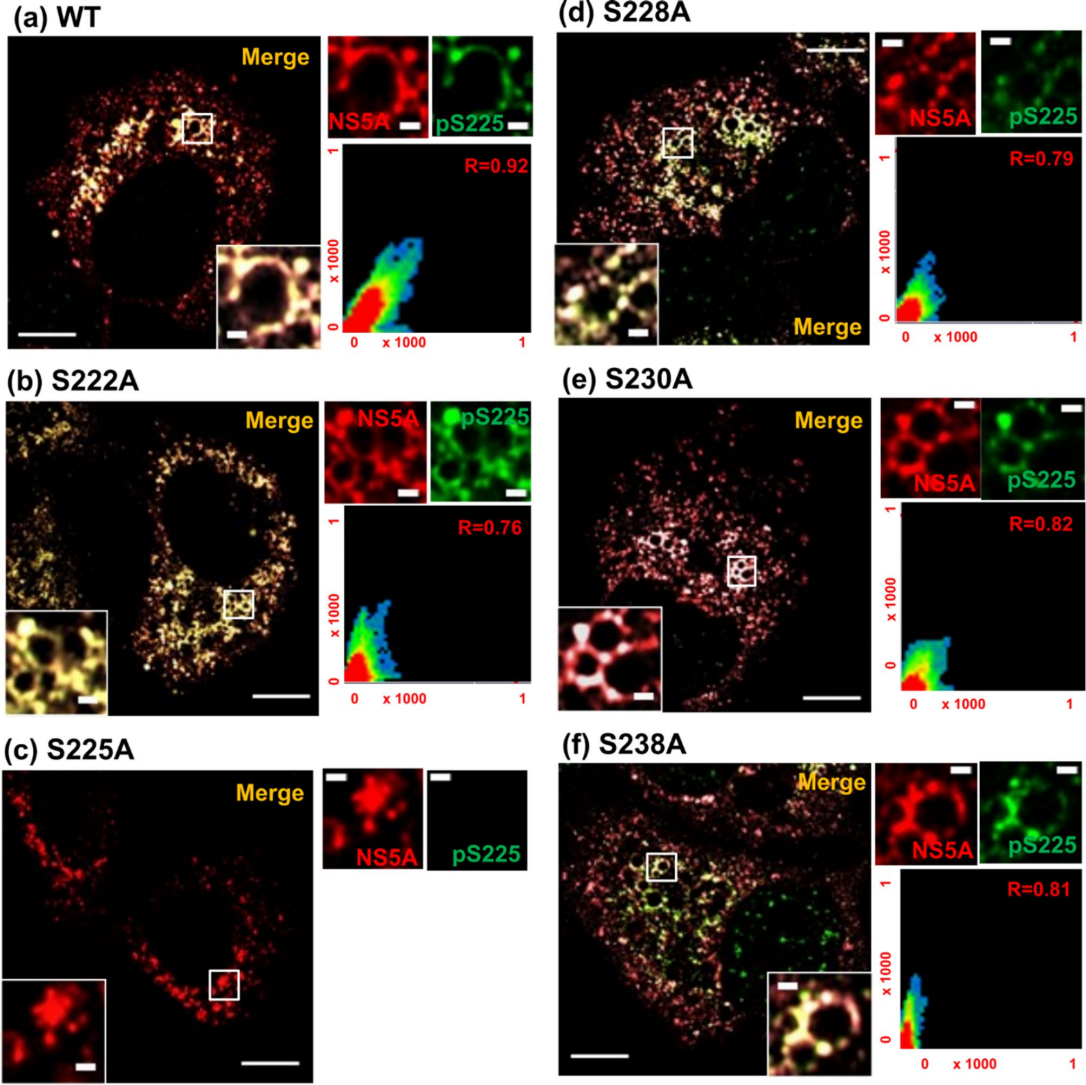
Subcellular distribution of phosphoablatant NS5A mutants. Huh7 cells were electroporated with the indicated mJFH-1 WT and mutant RNAs. At 48 h.p.e. cells were fixed and stained for total NS5A (red) and pS225 (green), prior to imaging by Airyscan microscopy. Scale bars: 10 μm and 1 μm (insets). Scatter plots show the co-localization of NS5A and LD (*x* and *y* axes, pixel numbers) performed on 10 cells taken from two independent experiments. The correlation value (*r*) is shown for each image.

**Fig. 4:**
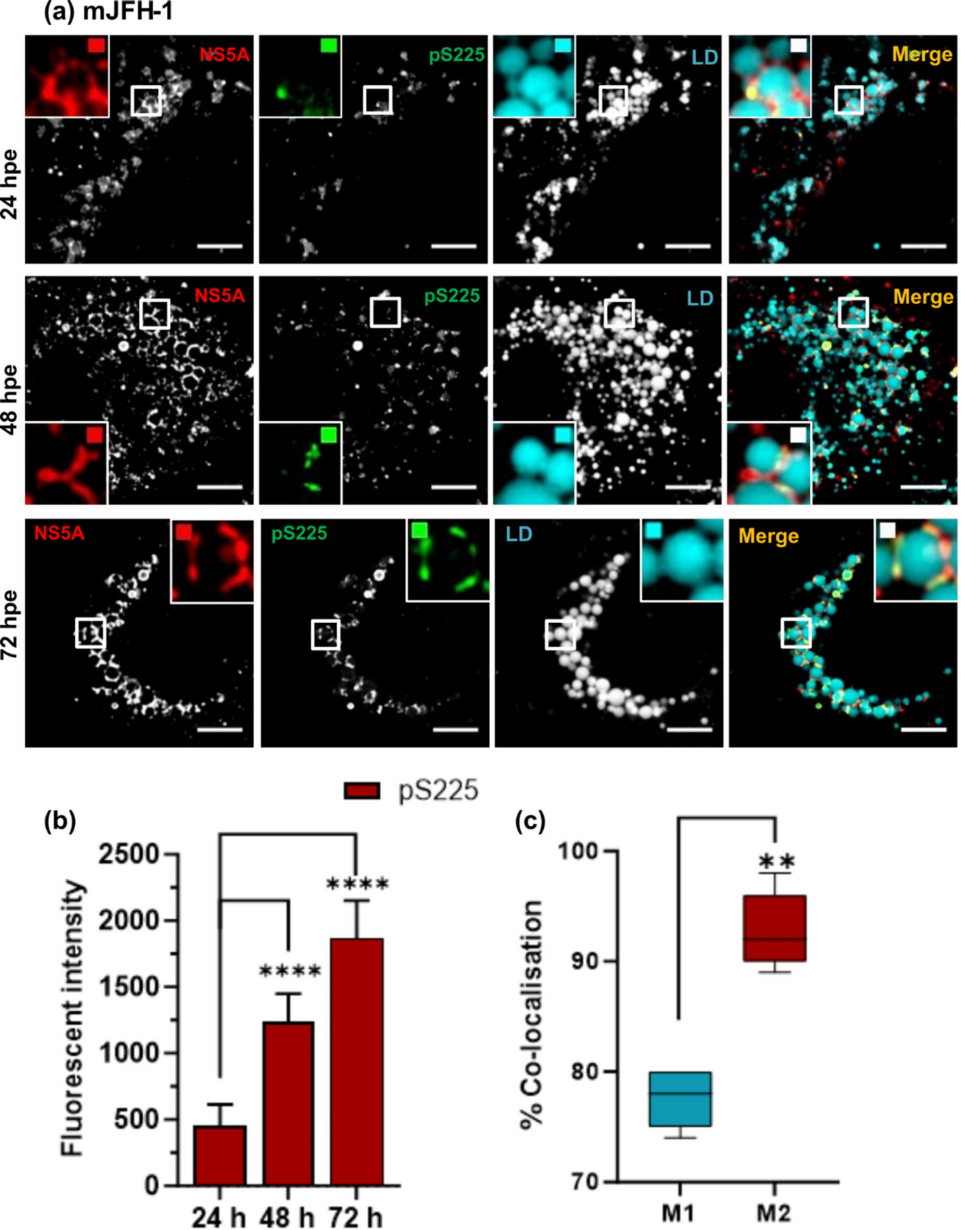
NS5A and pS225 subcellular distribution in HCV infected cells. (**a**) Huh7 cells were electroporated with WT mJFH-1 RNA, seeded onto coverslips and incubated for 24, 48 and 72 h. Cells were fixed and stained for total NS5A (red), pS225 (green) and LDs (Cyan) prior to imaging by confocal microscopy. Scale bars are 5 μm and 0.5 μm (insets). (**b**) Quantification of fluorescent intensity for pS225 was performed. Bar heights represent the mean of three technical repeats from two biological replicates, and error bars indicate ± 1 standard deviation. (**c**) Colocalisation analysis of LD and pS225. M1 indicates the percentage of the LD signal overlapping with the anti-pS225 signal, M2 the percentage of the anti-pS225 signal overlapping with the LD signal. Co-localisation calculations were performed on 10 cells from two independent experiments using Fuji ImageJ software with the Just Another Co-localisation Plugin (JACoP).

We next used confocal microscopy with Airyscan to analyse the distribution of both total NS5A and pS225-NS5A in cells infected with the various phosphoablatant mutants. As shown in Fig. 3, at 48 h.p.e. all mutants displayed a broad cytoplasmic distribution of NS5A that was comparable with wildtype, with the exception of S225A which exhibited a more compact perinuclear distribution, as previously demonstrated^23^. However, as observed in Fig. 1, we noted again that in all cases there was a subset of NS5A reactivity that did not co-stain with pS225. We thus undertook a temporal analysis of the distribution of total NS5A, pS225-NS5A and LDs in cells electroporated with mJFH-1 RNA (Fig. 4A). Quantification analysis revealed a significant increase in the amount of pS225 reactivity between 24 and 72 h.p.e (Fig. 4B). In addition at 72 h.p.e., the distribution of pS225 (Fig. 4C – co-efficient M2), and total NS5A, was highly associated with the periphery of LDs. As expected, no pS225 staining was observed in S225A control cells (Supp. Fig S3). Consistent with previous data^23,26,32^, this mutant showed a perinuclear distribution. Finally, the LDs in cells transfected with the GND mutant showed a broad cytoplasmic distribution and were less abundant than in wildtype infected cells at 72 h.p.e. (Supp. Fig S3).

### Expansion microscopy (ExM) imaging of NS5A and pS225 in HCV infected cells

Using stochastic optical reconstruction microscopy (dSTORM), we previously showed that the S225A phosphoablatant mutant NS5A was present in larger cytoplasmic puncta compared to wildtype or phosphomimetic S225D NS5A^23^. To investigate this further, we utilised expansion microscopy (ExM)^33–35^, which unlike other super-resolution approaches relies on the simple principle of physical expansion of the sample. Briefly, cells were probed with fluorescently labelled antibodies and embedded in a swellable gel matrix that chemically anchors the fluorescent labels. Samples were then physically expanded to ~ 4 times the original dimensions by swelling the gel in an aqueous environment. Using this method, ExM therefore improves diffraction-limited resolution by a factor of 4, achieving super-resolution (~ 70 –65 nm resolution). We applied this technique to interrogate pS225-NS5A and wt NS5A distribution in Huh7 cells.

**Fig. 5:**
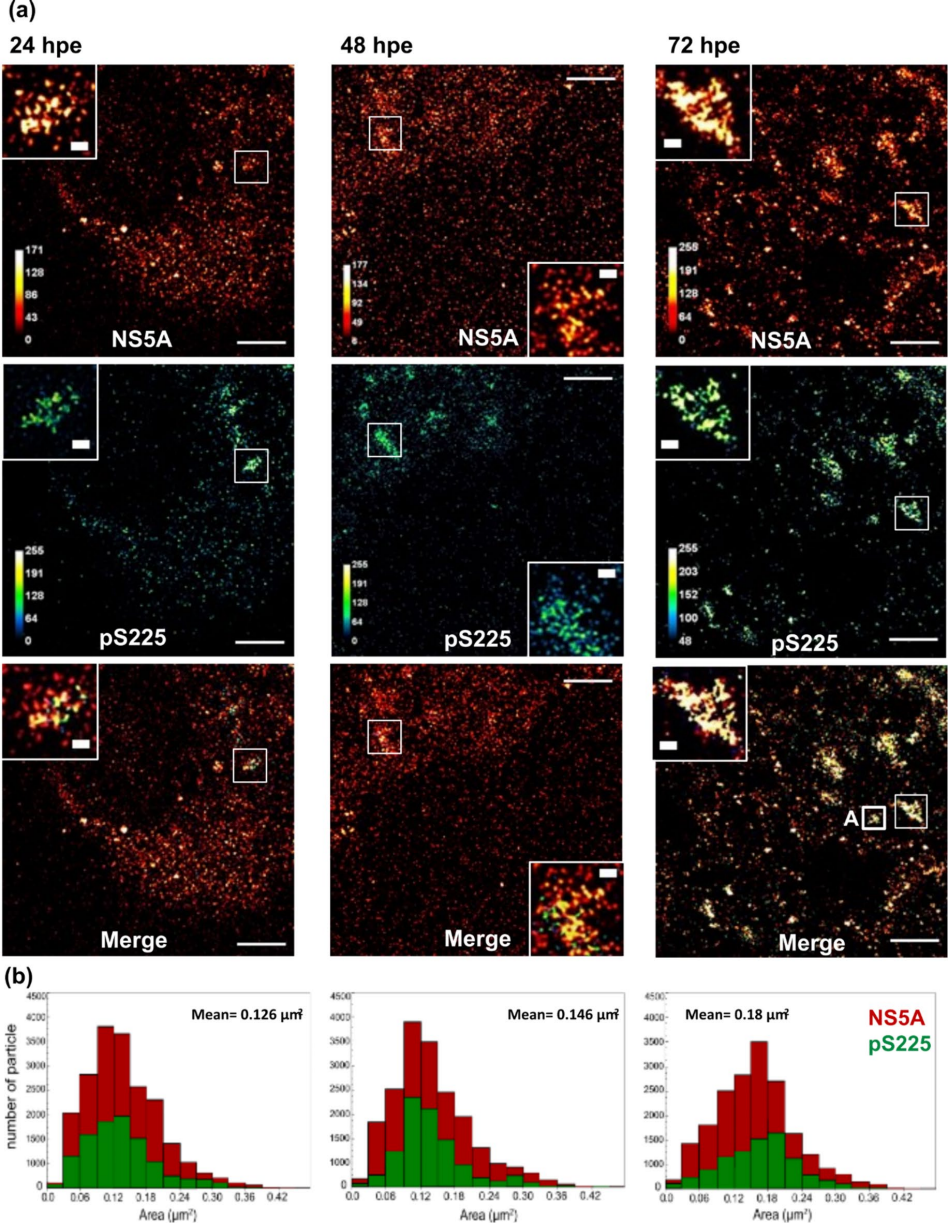
ExM analysis of HCV infected cells. (**a**) Huh7 cells were electroporated with WT mJFH-1 RNA, seeded onto 8-well glass chambers and incubated for 24, 48 and 72 h. Cells were fixed and stained for total NS5A (red) and pS225 (green), processed for ExM and imaged by confocal microscopy. Magnified views of boxed regions and individual channels are shown. Scale bars are 20 μm (physical size post-expansion factor, x4) and 1 μm (insets). Box A corresponds to the puncta that is subject to 3D-reconstruction in Fig. 7A. (**b**) Size of individual NS5A and pS225-NS5A punctae were determined and plotted as a frequency histogram. The area (µm^2^) is taken as an indication of the three-dimensional volume of the punctae. Mean values refer to the area of the pS225 punctae, the peaks labelled (1) and (2) illustrate the bimodal distribution of punctae.

Using Airyscan, ExM analysis revealed a diffuse distribution of NS5A throughout the cytoplasm (Fig. 5A), with significant co-localisation between NS5A and pS225 (merged panel). Over time, the diffuse distribution resolved into larger punctae with a peak area that increased from a mean of 0.126 µm^2^ at 24 h.p.e., to 0.18 µm^2^ at 72 h.p.e. (Fig. 5B).

**Fig. 6:**
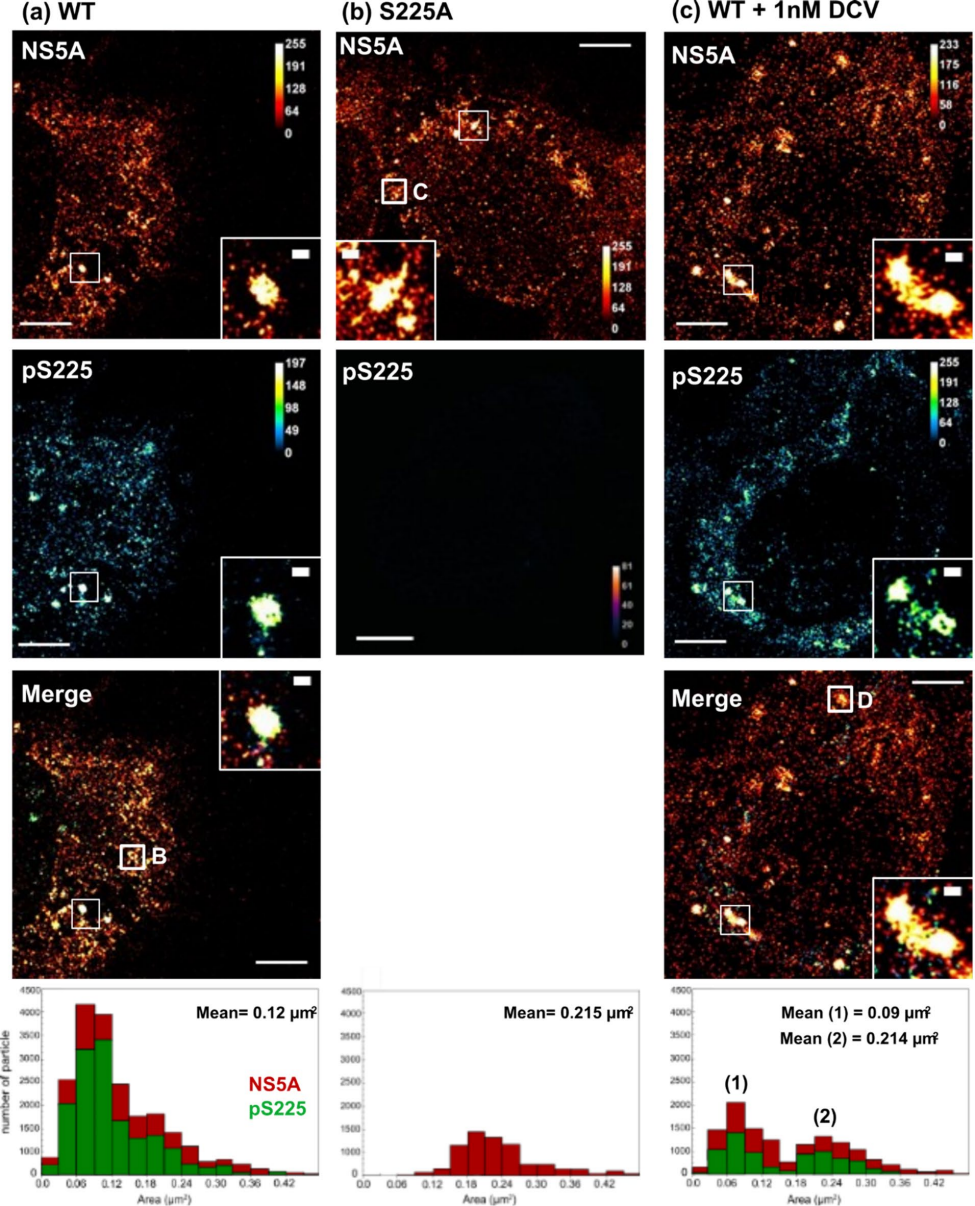
ExM analysis of SGR-harbouring cells. Huh7 cells harbouring either WT or S225A SGR were seeded onto 8-well chambered coverglass and incubated for 48 h. Cells were fixed and stained for total NS5A (red) and pS225 (green), processed for ExM, and imaged by confocal microscopy. Magnified views of boxed regions and individual channels are shown. Scale bars are 20 μm (physical size post-expansion factor, x4) and 1 μm (insets). (**a**) WT, (**b**) S225A and (**c**) WT following treatment with 1nM DCV for 24 h. Boxes B-D correspond to the punctae subject to 3D-reconstruction in Fig. 8A-C. The size of individual NS5A and pS225-NS5A punctae were determined and plotted as a frequency histogram (lower panels). The area (µm^2^) is taken as an indication of the three-dimensional volume of the punctae. Mean values refer to the area of the NS5A punctae.

For comparison, cells harbouring wildtype or S225A mutant SGR-Neo-JFH1 were also analysed by ExM. We reasoned that the lack of Core which interacts with the C-terminus of NS5A during virus assembly^36^ might affect the distribution of punctae. However this was not the case: as seen in the context of infectious virus wildtype NS5A was distributed throughout the cytoplasm (Fig. 6A), whereas S225A exhibited a concentration in the perinuclear region, albeit with a broader distribution as well (Fig. 6B). Interestingly, the proportion of NS5A puncta in wildtype SGR-harbouring cells that were pS225 positive appeared to be higher than for virus-infected cells although differences in methods preclude an absolute comparison (Fig. 6A green bars, compare to Fig. 4). Wildtype NS5A positive puncta were on average smaller (mean 0.12 µm^2^) than those in infected cells, however the S225A mutant exhibited significantly condensed and larger puncta (mean 0.215 µm^2^). It has been reported that treatment of HCV-infected cells with the NS5A inhibitor DCV results in a perinuclear accumulation of NS5A^12^, reminiscent of that observed for the S225A mutant. We therefore treated wildtype SGR harbouring cells with DCV and analysed the distribution of total NS5A and pS225-NS5A by ExM (Fig. 6C). Significant clustering of NS5A punctae were observed and size analysis revealed a bimodal distribution with two populations of punctae exhibiting small (mean 0.09 µm^2^) and large (mean 0.214 µm^2^) areas. The large population corresponded to the punctae observed in S225A mutant SGR harbouring cells.

**Fig. 7:**
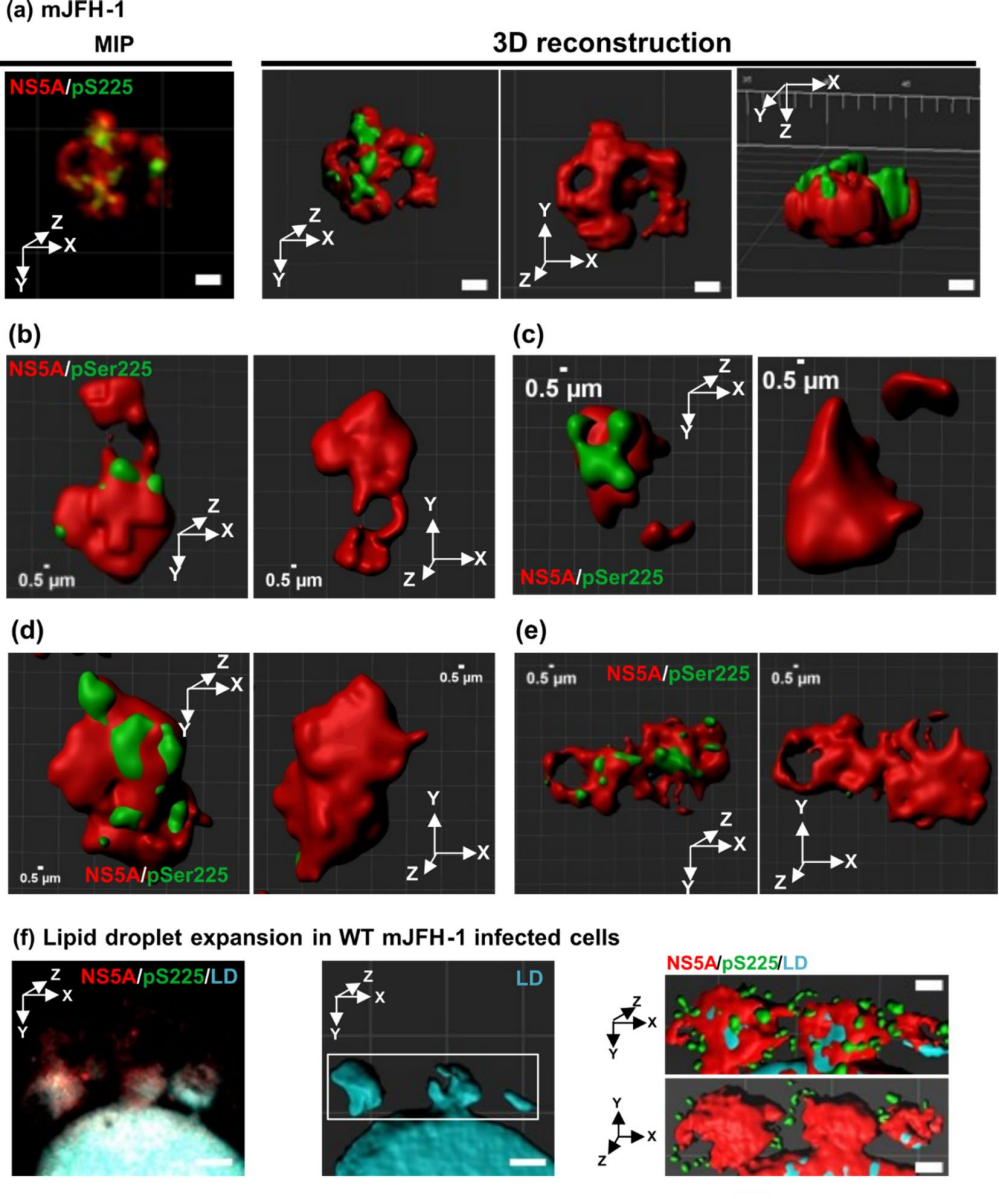
3D reconstruction of NS5A and pS225-NS5A punctae in mJFH-1 infected cells. (**a**) Left image shows a post-expansion maximum intensity projection (MIP). Right images show three-dimensional reconstructions of the NS5A puncta in 3 different orientations (as indicated by X, Y and Z arrows). (**b**-**e**) Further three-dimensional reconstructions of NS5A punctae in 2 different orientations (as indicated by X, Y and Z arrows) from cells infected with mJFH-1. Scale bars 0.5 μm. (**f**) LD expansion from cells infected with mJFH-1. LDs were stained with anti-ADRP (cyan). The left image shows a MIP, the central image shows a three-dimensional reconstruction of ADRP staining, right panel shows two orientations of the punctae in the white box. Scale bars in A and F: 1 μm.

To fully exploit the improved resolution afforded by ExM, we next performed a 3D-reconstruction of individual NS5A-positive punctae. We analysed punctae from mJFH-1-infected cells (shown in Fig. 5A, box A), wildtype SGR-harbouring cells (Fig. 6A, box B), S225A SGR-harbouring cells (Fig. 6B, box C) or wildtype SGR-harbouring cells treated with DCV (Fig. 6C, box D). In Fig. 7A, the left panel shows the maximum intensity projection (MIP) of an individual puncta from an mJFH-1 infected cell used to calculate localisation volumes and to generate three-dimensional (3D) reconstructions, followed by 3 different profiles of the puncta (top, bottom and side). Figure 7B-E and Supp. Fig S4 show more examples of these 3D reconstructions, in addition a video of the puncta in Fig. 7E is presented in Supp. Fig S4E. In mJFH-1 infected cells pS225 was found in distinct areas on the surface of the punctae, mostly adjacent to cavities that extended through the punctae. The diameter of these cavities varied from 0.17 to 0.4 μm, consistent with LDs. Technical limitations of ExM mean that the BODIPY (558/568)-C_12_ dye cannot be anchored to the gel matrix and that LDs cannot be directly visualised. To further investigate the localisation of the pS225 positive areas, we therefore stained mJFH-1 infected cells with an antiserum to the cellular LD-associated protein, adipocyte differentiation-related protein (ADRP)^22,37^ prior to processing for ExM (Fig. 7F). This analysis revealed a partial co-localisation of pS225 with LDs and that NS5A punctae surround these LDs.

**Fig. 8:**
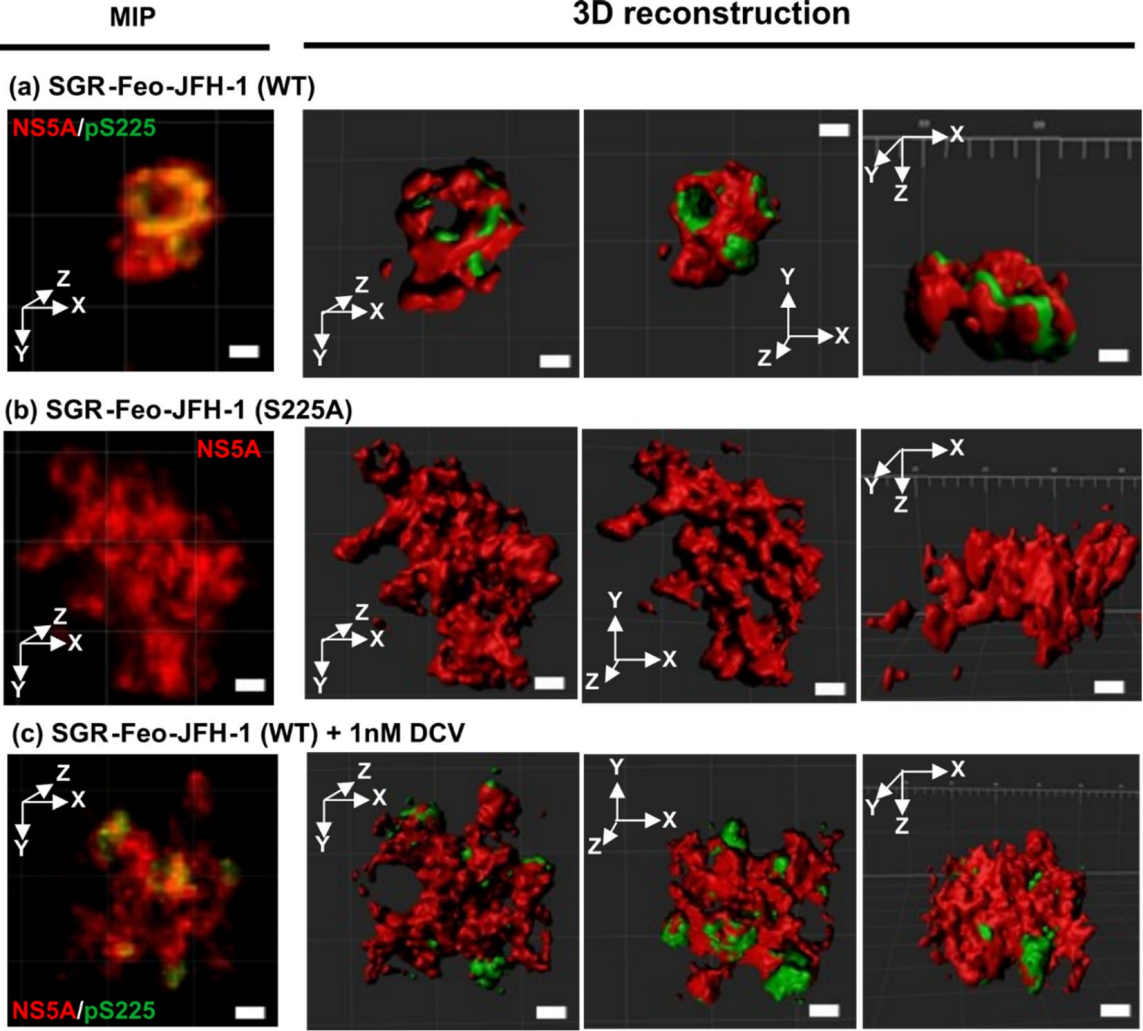
3D reconstruction of NS5A and pS225-NS5A punctae in SGR harbouring cells. (**a**) SGR-Feo-JFH-1 (WT), (**b**) SGR-Feo-JFH-1 (S225A) and (**c**) SGR-Feo-JFH-1 (WT) following treatment with 1nM DCV for 24 h. Scale bars: 1 μm.

In SGR-harbouring cells (Fig. 8A), pS225 was more widely distributed over the surface of NS5A punctae, remaining close to cavities. The compact structure of the punctae was not observed in S225A mutant SGR-harbouring cells, in which NS5A occupied larger irregular-shaped punctae and spaces that likely represent LDs (Fig. 8B). Intriguingly, NS5A punctae in DCV-treated cells exhibited the same expanded, irregular structures observed for S225A with pS225 distributed across these punctae often at the edges (Fig. 8C).

## Discussion

Despite extensive studies, the functions of NS5A remain to be unambiguously defined. This issue was brought into focus by the advent of DAAs that are predicted to target NS5A. These compounds are highly effective at inhibiting HCV replication, both in vitro and in patients, yet virtually nothing is known about their mode of action, or the functions of NS5A that they inhibit. In this regard, a growing body of evidence points to phosphorylation as a key regulator of NS5A function, although both biochemical and cell biological investigations of NS5A phosphorylation remain inconclusive. Our studies have focused on phosphorylation of S225 in the serine-rich LCSI between DI and DII. Here, we describe the use of a unique antiserum specific for pS225 that has allowed us for the first time to directly interrogate the role of S225-phosphorylated NS5A.

Use of this antiserum first revealed that pS225 was predominantly present within the p58 NS5A species. We previously observed an extensive phenotype for the S225A mutant^23,31^ and considered this a disproportionate effect of a single serine-alanine substitution in a highly serine-rich region (Fig. 1A). This prompted us to consider that S225 phosphorylation drives additional proximal phosphorylation events in a sequential (or hierarchical) fashion. Our data are consistent with this (Fig. 2), and we propose a model whereby S225 phosphorylation primes subsequent phosphorylation in a bidirectional manner. Confirmation of this model would require use of additional phospho-specific antibodies, such as those used recently to demonstrate that pS232 primes phosphorylation at S235 and S238^27,28^, an observation consistent with our findings. We propose that phosphorylation of S225 leads to multiple phosphorylation events within a short sequence (17 amino acids), producing a closely packed cluster of phosphates whose negative charge has implications for both protein-protein interactions and conformation, for example by changing the orientation of DI and DII respective to each other.

We next combined the pS225 antiserum with two super-resolution microscopy approaches (Airyscan and ExM) which can overcome the light diffraction limit of conventional microscopy to interrogate the subcellular distribution of pS225 in comparison to the total pool of NS5A. This revealed that pS225-NS5A was only a subset of the total, consistent with the presence of both p56 (lacking pS225) and p58 (pS225-positive) species (Fig. 1B). However, we observed that pS225 was not uniformly distributed across the total pool of NS5A (Fig. 4). Three-dimensional reconstruction of NS5A punctae imaged by ExM revealed that pS225 foci were surface exposed and in many cases were close to cavities that extended through the punctae (Fig. 7). Staining with an antiserum to ADRP suggested that these cavities likely represent LDs and were in some cases surrounded by NS5A. However, these data are difficult to unambiguously interpret for technical reasons. We were only successful in expanding either small LDs or protrusions from large LDs. ExM is dependent on many factors including the physical properties of sub-cellular structures such as size and the presence of a limiting monolayer or bilayer. To overcome these limitations, ExM will require further technical advances. In summary, although it is plausible that the cavities observed in juxtaposition to a subset of pS225-NS5A represent LDs, it is also possible that they are occupied by other cellular factors.

These observations lead to a number of speculative conclusions. Firstly, the location of pS225 near to LDs is consistent with the loss of the NS5A: LD association following treatment with kinase inhibitors^38^, and suggests that pS225 is required for the stable interaction of NS5A with LDs. It is conceivable that the concentration of phosphorylated serines in LCSI mediates interactions with lipids on the surface of LDs. There is precedent for this: for example during lipolysis, cytosol to LD translocation of hormone sensitive lipase (HSL) is driven by phosphorylation of two adjacent serines by protein kinase A. The LD-resident protein perilipin is also extensively phosphorylated during this process^39^. Secondly, the surface location of pS225-NS5A would facilitate its interactions with other cellular factors explaining the marked effect of S225A^23^ on the distribution of NS5A. Indeed it is implicit that S225-phosphorylated NS5A would be present on the surface of punctae, as only surface located NS5A molecules would be accessible for phosphorylation by cellular kinases. As the punctae likely represent sites of genome replication which would presumably occur within the punctae (thus protected from cytosolic dsRNA sensors), this may also imply that pS225-NS5A is not directly involved in genome replication but instead plays other roles in trafficking of replication complexes or targeting to (for example) LD. ExM also allowed us to observe dramatic alterations in the architecture of NS5A punctae resulting from either the S225A phosphoablatant mutation, or DCV treatment of wildtype (Fig. 8). The compact and smooth architecture of the wildtype NS5A punctae with cavities (likely to be LDs) extending through the structure was replaced with an expanded and irregular structure with less distinct cavities. The similarities suggest that S225 phosphorylation and DCV regulate common function(s) of NS5A, possibly protein-protein and/or protein-lipid interactions. This is consistent with previous studies showing that DCV treatment disrupts these structures and distribution and formation of membrane-associated replication complexes^40,41^. As a note of caution, these previous studies were performed with NS3-5B expression constructs, rather than in the context of HCV infection.

In conclusion, we provides further evidence supporting the critical role of phosphorylation in regulating the function of NS5A. In particular, we propose that S225 phosphorylation is the primary site within LCSI that primes subsequent phosphorylation events. The high degree of conservation of this residue, and the adjacent serines in LCSI, in all genotypes of HCV suggests some common mechanism across HCV isolates. However, it is intriguing that in the closely related *Hepacivirus equi*^42^ the LCSI cluster of serines is conserved but the residue corresponding to S225 is aspartate (D). S225D is a phosphomimetic substitution, mimicking the negative charge of the phosphate that may maintain the functionality of pS225. Future comparative studies between different hepaciviruses may help to elucidate the role(s) of phosphorylation in regulating NS5A function.

## Methods

### Plasmids

DNA constructs mSGR-Feo-JFH-1 and mJFH-1 containing unique restriction sites flanking the NS5A coding sequence^43^ were derived from the full-length pJFH-1 virus^44^, and the luciferase-reporter containing sub-genomic replicon (pSGR-luc-JFH-1) constructs^45^. The serine-alanine mutants have been described previously^26^.

### Anti-pS225 Sera

Rabbits were immunised with the 14-mer peptide CARGSPP**pS**EASSS conjugated to KLH. IgG was depleted using the non-phosphorylated peptide and purified by enrichment using the phospho-peptide (DC Biosciences, Dundee).

### Cell culture

Huh7 cells were obtained from Ralf Bartenschlager (University of Heidelberg) and were maintained in Dulbecco’s modified Eagle’s medium (DMEM; Sigma-Aldrich) supplemented with 10% foetal bovine serum (FBS), 100 IU penicillin/ml, 100 µg streptomycin/ml, and 1% non-essential amino acids (NEAA) in a humidified incubator at 37 °C with 5% CO_2_. Huh7 cells stably harbouring a subgenomic JFH-1 replicon (SGR-Feo-JFH-1) were maintained in the same medium supplemented with G418 (300 µg/ml). All cells were checked monthly for mycoplasma contamination.

### Electroporation

Huh7 cells (4 × 10^6^) were washed twice in PBS, re-suspended in PBS and electroporated with 2 µg of RNA at 975 µF and 260 V. Cells were resuspended in complete media and plated in 96-well (3 × 10^4^cells/well), or 6-well plates (3 × 10^5^ cells/well).

### SDS-PAGE/Western blot

Cells were washed twice in PBS, lysed in GLB (1% (v/v) Triton X-100, 120 mM KCl, 30 mM NaCl, 5 mM MgCl_2_, 10% (v/v) glycerol, 10 mM PIPES-NaOH, pH 7.2 with protease and phosphatase inhibitors) and harvested by centrifugation (2800 x g, 10 min, 4 °C). Following SDS-PAGE, proteins were transferred to a polyvinylidene difluoride (PVDF) membrane and blocked in 50% (v/v) Odyssey blocking (OB) buffer (LI-COR) in TBS. Membranes were labelled with primary antibodies overnight at 4 °C, followed by secondary antibodies for 2 h at room temperature (RT), both prepared in 25% OB buffer. Primary antibodies were anti-NS5A (sheep, 1:4,000)^46^, anti-pS225 (1:1000) and mouse α-Actin (Sigma, 1:10,000). Secondary antibodies were anti-rabbit, anti-sheep (800 nm) or anti-mouse (700 nm), used at 1:10,000 prior to imaging using a LI-COR Odyssey Sa infrared imaging system. Quantification was performed using Image Studio v3.1 (LI-COR) using a background subtraction method.

### Immunofluorescence and confocal microscopy

Cells were washed with PBS, fixed for 20 min in 4% (w/v) PFA, permeabilised in PBS/0.1% (v/v) Triton X-100 (PBS-T), blocked with PBS-T/5% (w/v) BSA before staining with primary antibody (sheep anti-NS5A 1:2000, mouse anti-NS5A; 1:1000, rabbit anti-pS225; 1:1000, sheep-anti ADRP 1:1000). Fluorescently conjugated secondary antibodies were used at 1:500 (Life Technology). Nuclei were counterstained with DAPI. Lipid droplets were stained with BODIPY (558/568)-C12 (1:1,000, Life Technology). Confocal images were acquired on a Zeiss LSM880 microscope with Airyscan, post-acquisition analysis was conducted using Zen software (Zen version 2015 black edition 2.3, Zeiss) or Fiji ImageJ (v1.49) software^47^.

### Expansion microscopy (ExM)

Cells were fixed and stained as above, prior to treatment with 0.1 mg/ml Acryloyl-X, SE (6-((acryloyl)amino) hexanoic acid, succinimidyl ester (AcX) in PBS, (stock 10 mg/ml in DMSO), overnight at RT. For gelation, ammonium persulfate (APS) and tetramethylethylenediamine (TEMED) (0.2% (w/w) each) were added to the monomer solution (1x PBS, 2 M NaCl, 8.625% (w/w) sodium acrylate, 2.5% (w/w) acrylamide, 0.15% (w/w) N, N′- methylenebisacrylamide). The inhibitor 4-hydroxy-2,2,6,6-tetramethylpiperidin-1-oxyl (4-HT) (0.01% (w/w)) was also added. Gelation was performed for 30 min at RT in the same 8 well chamber glass slides, placing a non-adhesive glass slide over the top of the gasket to seal the chamber. Digestions were performed by incubating gels on a rocker overnight at RT with proteinase K (16 units/mL in 50 mM Tris-HCl pH 8.0, 1 mM EDTA, 0.5% Triton X-100, 0.5 M NaCl). Gels were expanded in excess volumes of double-deionized water for 30 min, replacing the water for additional 30-min washes 4 times.

### Three-dimensional image acquisition

The samples were imaged with Zeiss LSM880 microscope, using a plan-Apochromat 63x/1.4 Oil DIC, and a 405-nm diode laser, 488 nm line of argon laser, 561-nm DPSS diode laser, 633-nm HeNe laser: with 70-nm *xy* pixel size, and 210-nm *z*-spacing. Image stacks were saved as series of 16-bit TIFF files for further processing.

The acquired image stacks were processed using Zeiss Black software and the maximum-likelihood estimation (MLE) algorithm was selected for deconvolution. Deconvolved image volumes were re-sampled onto 50 × 50 × 50 nm voxel grids in 32-bit TIFF format. The binary image volumes were used for generating a 3D skeleton in Amira 5.4 (Visage Imaging, Berlin, Germany).

### Co-localisation analysis

Manders’ overlap coefficients were calculated using Fiji ImageJ software with Just Another Co-localisation Plugin (JACoP) (National Institutes of Health). Co-localisation calculations were performed on 10 cells from at least three independent experiments.

## Electronic supplementary material

Below is the link to the electronic supplementary material.

Supplementary Material 1

## Data Availability

The datasets generated during and/or analysed during the current study are available from the corresponding author on reasonable request.
