# Supplementary material for: A key role for hepatitis C virus NS5A serine 225 phosphorylation revealed by super-resolution microscopy
Source: Sci Rep. 2025 Mar 20;15:9567. doi: 10.1038/s41598-025-93812-w (PMC11926191; doi:10.1038/s41598-025-93812-w)
Supplement: Supplementary file 1 — Supplementary Material 1 [file 41598_2025_93812_MOESM1_ESM.pptx]

## Slide 1
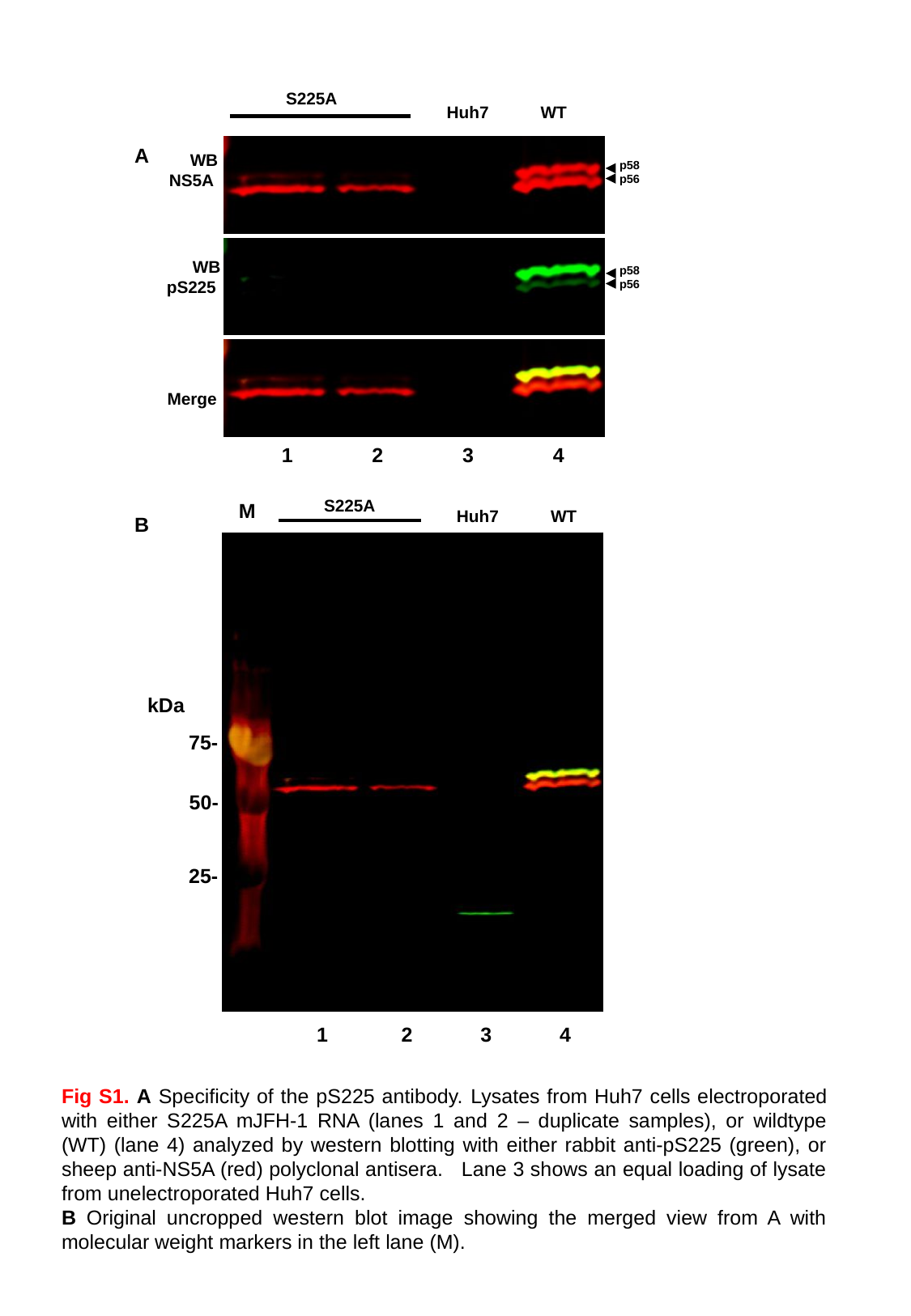

S225A
WT
Huh7
A
WB
NS5A
p58
p56
WB
pS225
p58
p56
Merge
1 2 3 4
S225A
M
WT
Huh7
B
kDa
75-
50-
25-
1 2 3 4
Fig S1. A Specificity of the pS225 antibody. Lysates from Huh7 cells electroporated with either S225A mJFH-1 RNA (lanes 1 and 2 – duplicate samples), or wildtype (WT) (lane 4) analyzed by western blotting with either rabbit anti-pS225 (green), or sheep anti-NS5A (red) polyclonal antisera. Lane 3 shows an equal loading of lysate from unelectroporated Huh7 cells.
B Original uncropped western blot image showing the merged view from A with molecular weight markers in the left lane (M).

## Slide 2
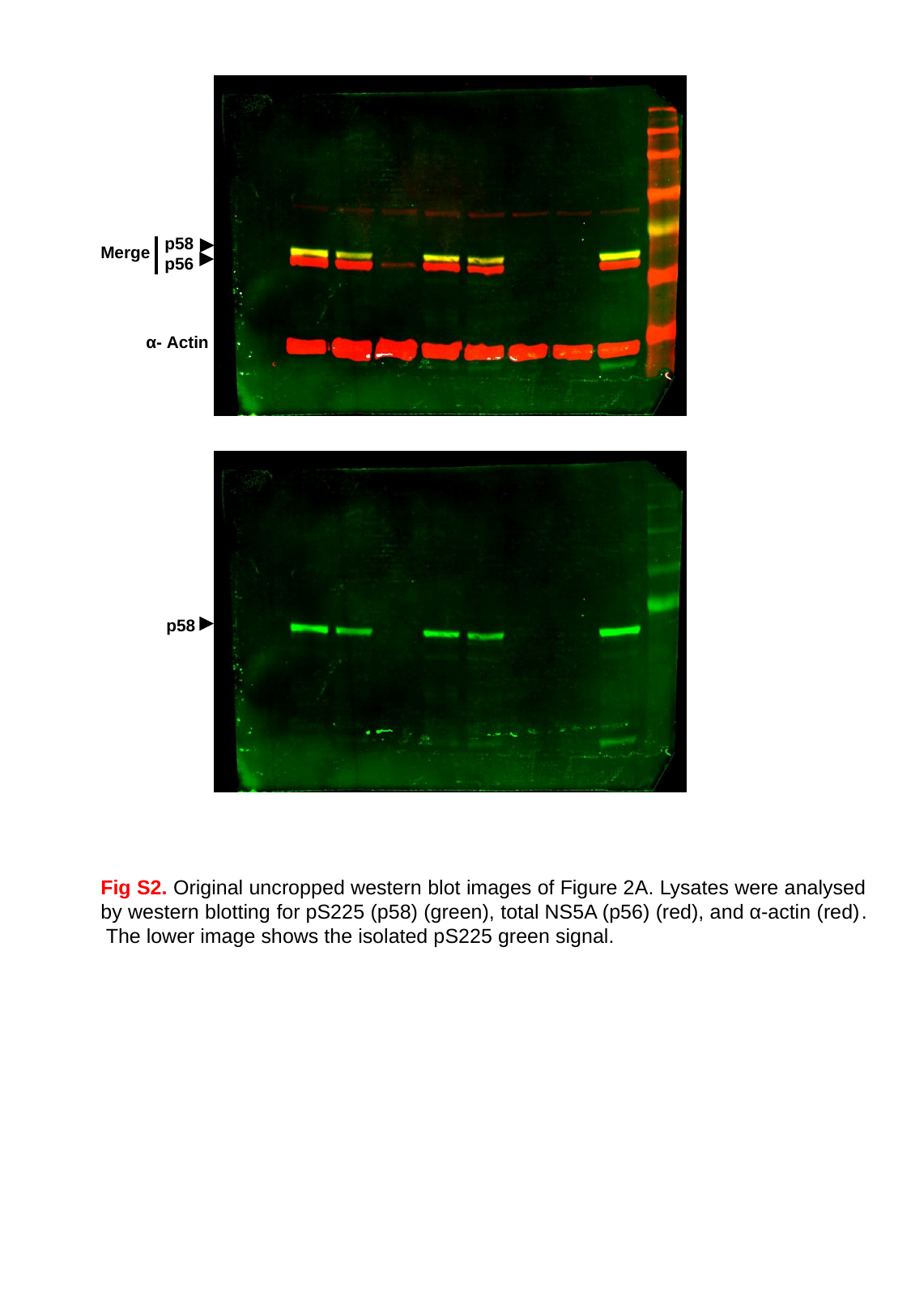

p58
p56
Merge
α- Actin
p58
Fig S2. Original uncropped western blot images of Figure 2A. Lysates were analysed by western blotting for pS225 (p58) (green), total NS5A (p56) (red), and α-actin (red). The lower image shows the isolated pS225 green signal.

## Slide 3
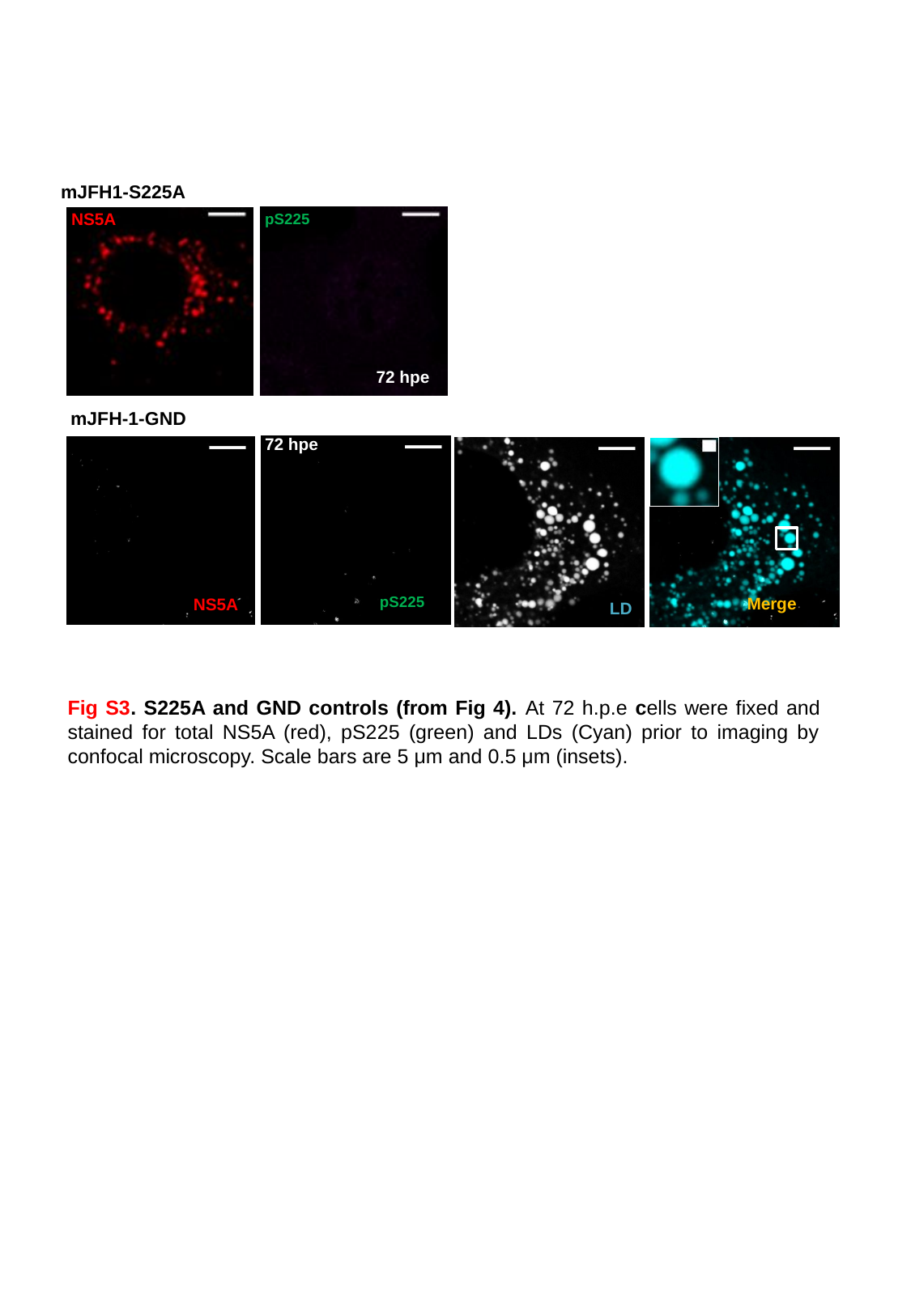

mJFH1-S225A
NS5A
pS225
72 hpe
mJFH-1-GND
72 hpe
pS225
pS225
Merge
NS5A
LD
Fig S3. S225A and GND controls (from Fig 4). At 72 h.p.e cells were fixed and stained for total NS5A (red), pS225 (green) and LDs (Cyan) prior to imaging by confocal microscopy. Scale bars are 5 μm and 0.5 μm (insets).

## Slide 4
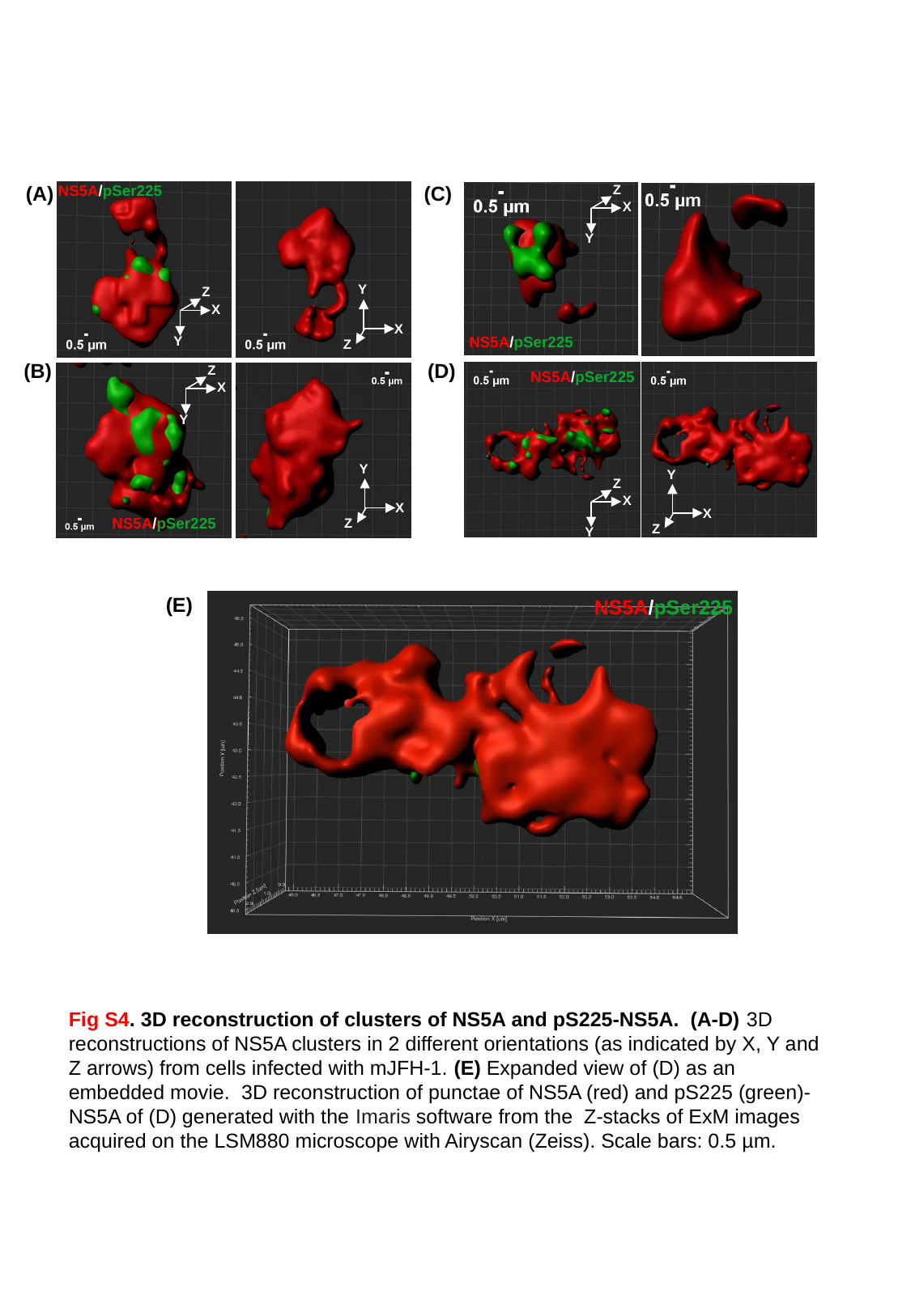

(A)			 (C)
Z
X
Y
NS5A/pSer225
Y
X
Z
Z
X
Y
Z
X
Y
Y
X
Z
NS5A/pSer225
NS5A/pSer225
(B)			 (D)
NS5A/pSer225
Y
X
Z
Z
X
Y
(E)
NS5A/pSer225
Fig S4. 3D reconstruction of clusters of NS5A and pS225-NS5A. (A-D) 3D reconstructions of NS5A clusters in 2 different orientations (as indicated by X, Y and Z arrows) from cells infected with mJFH-1. (E) Expanded view of (D) as an embedded movie. 3D reconstruction of punctae of NS5A (red) and pS225 (green)-NS5A of (D) generated with the Imaris software from the  Z-stacks of ExM images acquired on the LSM880 microscope with Airyscan (Zeiss). Scale bars: 0.5 µm.
